# Ureter as an Innocent Bystander: Presentation and Management in Unusual Vascular Compression Syndromes

**DOI:** 10.7759/cureus.86580

**Published:** 2025-06-23

**Authors:** Harkirat Talwar, Vikas K Panwar, Tushar A Narain, Ankur Mittal

**Affiliations:** 1 Urology, Max Super Speciality Hospital, Noida, IND; 2 Urology, All India Institute of Medical Sciences, Rishikesh, IND; 3 Urology, Max Smart Super Speciality Hospital, Saket, New Delhi, IND

**Keywords:** common iliac artery, gonadal vessels, inferior mesenteric vein, ureter, ureteric compression, vascular compression

## Abstract

Objective

Abdominopelvic vascular compression syndromes occur when the vascular structures either cause compression or are compressed by the surrounding hollow viscera. Apart from retrocaval ureter and UPJO (ureteropelvic junction obstruction), ureteric compression by other vascular structures is rare. We present five rare cases of ureter compression caused by the inferior mesenteric vein, testicular vein, ovarian vein, common iliac arteries, and an unnamed tributary of the inferior vena cava (IVC).

Methods

Retrospective data of all cases of hydroureteronephrosis between January 2019 and March 2020 were studied. Out of the 659 cases identified, the search was narrowed to keywords like "vascular compression", "ureteric compression", and "crossing vessels". A total of 11 cases were identified. Excluding six cases of UPJO, we were left with five cases of extrinsic ureteric compression caused by other vascular structures.

Results

Case 1 was a 26-year-old man with ureteric compression by the inferior mesenteric vein. Case 2 was a 27-year-old man with an incidental intraoperative finding of the left testicular vein compressing the upper ureter. Case 3 was a 38-year-old female with a dilated upper ureter due to compression by the right ovarian vein. Case 4 was a 19-year-old female with compression of bilateral mid-ureters by common iliac arteries. Case 5 was a 26-year-old man with an upper ureteric stricture due to a crossing tributary of the IVC.

Conclusion

Vascular compression of the ureter, by ovarian or testicular veins, common iliac arteries or veins, or unusual IVC tributaries, though rare, can cause proximal hydroureteronephrosis, stones, and pyelonephritis. CT urography, which includes arterial, venous, and excretory phases, is essential for accurate diagnosis. Management of such syndromes includes retrograde pyelography +/- temporary stenting and definitive minimally invasive measures like endoscopic ureterotomy, ureterolysis, and/or definitive reconstruction, effectively relieving obstruction and preventing complications.

## Introduction

The ureter is a hollow, pliable tube that can easily get compressed by fixed abdominal structures. Apart from the inferior vena cava (IVC) in the retrocaval ureter and the crossing vessels in ureteropelvic junction obstruction (UPJO), ureteric compression by other vascular structures in the abdomen, causing upstream hydroureteronephrosis and dilatation of the pelvicalyceal system (PCS), is a very rare phenomenon. Several abdominopelvic vascular compression syndromes are well-known in the literature, in which the vascular structures either cause compression or are compressed by the surrounding hollow viscera. Some of the more well-known of them are median arcuate ligament syndrome (MALS), May-Thurner syndrome, nutcracker syndrome, and superior mesenteric artery (SMA) syndrome [1].

These syndromes present with varied symptoms, depending on the viscera or vessel being compressed, and are usually vague and nonspecific. Frequently, patients remain asymptomatic, and such syndromes are diagnosed incidentally on imaging for unrelated causes [1].

This study aimed to underscore five exceptionally rare causes of ureteric obstruction due to extrinsic vascular compression by the inferior mesenteric vein (IMV), testicular vein, ovarian vein, common iliac arteries, and an unnamed tributary of the IVC. Notably, the compression caused by the IMV, resulting in upstream hydroureteronephrosis, appears to be a previously unreported occurrence, representing the first documented case of its kind in the literature.

## Materials and methods

Study design

This is a retrospective study conducted in our department, aimed at determining the incidence of unusual vascular compression syndromes of the ureter that cause upstream hydroureteronephrosis. The study obtained approval from the Institutional Ethics Committee of All India Institute of Medical Sciences (AIIMS) (approval no: AIIMS/IEC/20/431). Retrospective data of all cases of hydronephrosis and hydroureteronephrosis between January 2019 and March 2020 were studied. We utilized the hospital's Radiology Information System (RIS)/PACS, which included keyword filters ("hydroureteronephrosis", "hydronephrosis"), implemented via the RIS's built-in query tool. Out of the 659 cases identified, we narrowed our search to keywords like "vascular compression", "ureteric compression", and "crossing vessels". A total of 11 such cases were identified. Excluding six cases of UPJO, which had crossing vessels, we were left with five cases of extrinsic ureteric compression caused by other vascular structures.

Inclusion criteria

All cases of hydronephrosis or hydroureteronephrosis confirmed to be caused by extrinsic vascular compression on the ureter, regardless of patient age or sex, were included in the study. Compression must originate from vascular structures external to the ureter, such as ovarian/testicular veins, the IMV, the common iliac artery, and the IVC tributaries.

Exclusion criteria

Cases of UPJO attributed solely to intrinsic or accessory lower-pole renal crossing vessels, such as segmental arteries or veins crossing at the ureteropelvic junction (UPJ), were excluded from the study.

No statistical analysis was employed during the study. As a single-center, retrospective case series, our inclusion relied on available records of hydroureteronephrosis cases with documented vascular compression. We recognize that not all eligible cases may have been captured, and this may introduce selection bias. To mitigate this, we applied strict inclusion and exclusion criteria and conducted two independent record reviews to ensure consistency.

## Results

Case 1

**Figure 1:**
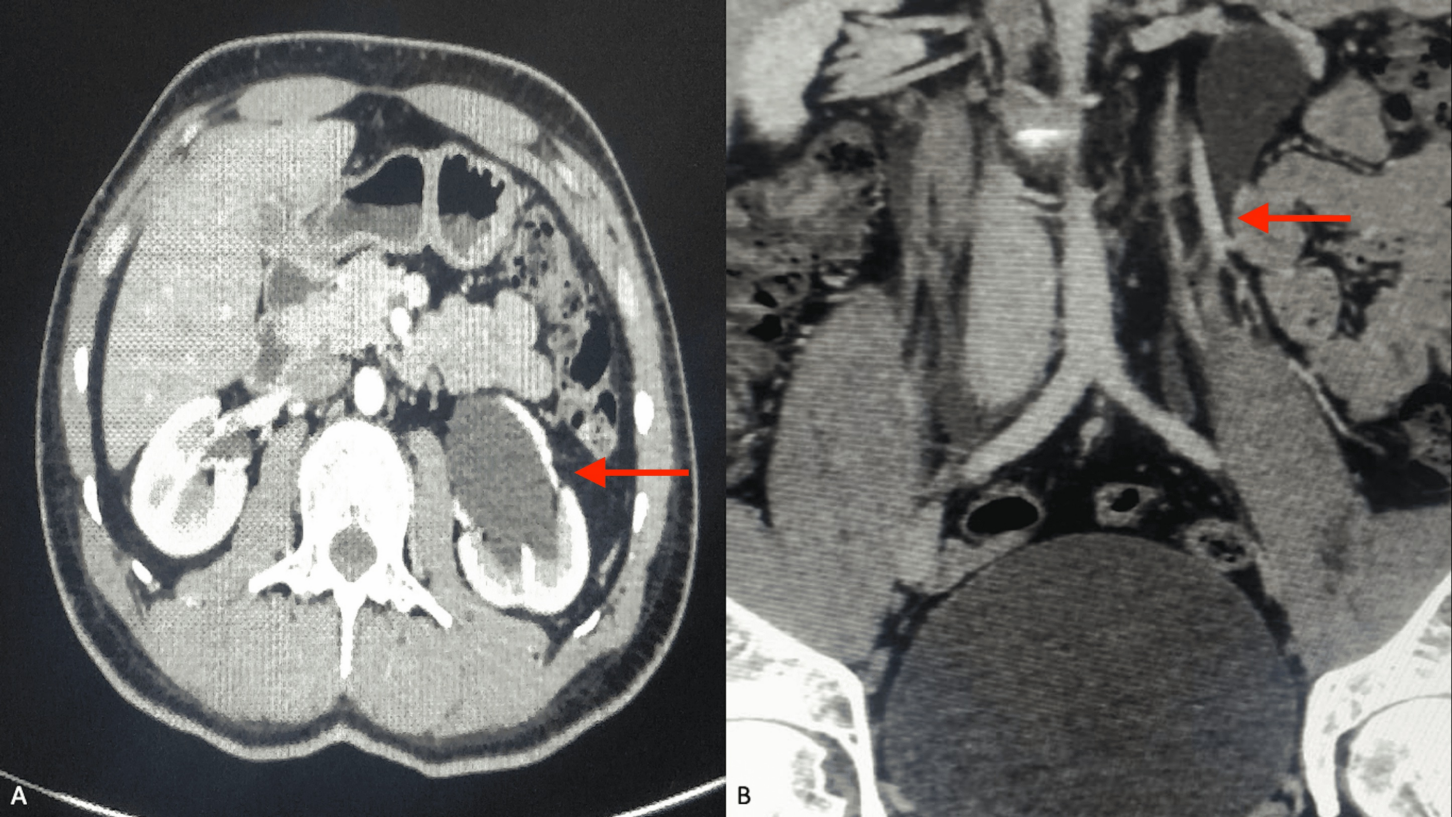
Multidetector CT scan showing compression of the left ureter by the crossing IMV (B), causing upstream hydroureteronephrosis (A) IMV: inferior mesenteric vein

A young male, 26 years of age, presented to the primary caregiver with complaints of left flank pain for three weeks. The pain was continuous, with a dull aching character, and increased in intensity a few minutes to hours after ingestion of food, then decreased thereafter. There were no other complaints. His physical examination and vitals were unremarkable. Blood and urine laboratory workup was within normal limits. An abdominal ultrasound showed moderate hydronephrosis on the left side with a normal right kidney. A contrast-enhanced CT scan revealed the IMV crossing over and compressing the left upper ureter with consequent upstream hydroureteronephrosis (Figure 1).

**Figure 2:**
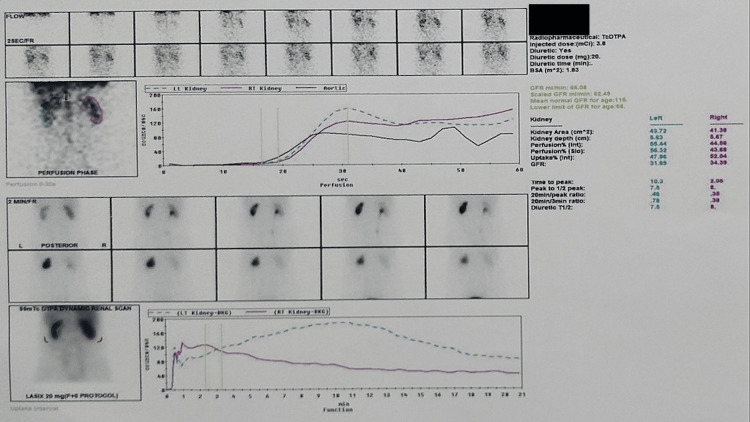
Tc-99 DTPA renogram with diuretic showing normal parenchymal function with sluggish drainage of the left hydronephrotic kidney Tc-99m DTPA: technetium-99m diethylenetriaminepentaacetate

Further, a nuclear diuretic scan (Tc-99 DTPA (technetium-99m diethylenetriaminepentaacetate) renogram with diuretic) was performed, which showed a hydronephrotic left kidney with normal parenchymal tracer uptake with sluggish drainage, although there was no contrast retention on delayed images (Figure 2). The right kidney showed normal parenchymal function and drainage. The time to peak was 10.3 minutes on the left side as compared to 2.08 minutes on the right side. Thus, sluggish drainage was confirmed on the left side by the nuclear scan, thought to be due to ureter compression by the IMV. The patient underwent a left endoscopic ureteric stent placement as he was symptomatic. His symptoms resolved, and the stent was removed after four weeks. At the four-month follow-up CT scan, hydroureteronephrosis was persistent, and a left ureteroureterostomy/pyelostomy is planned.

Case 2

**Figure 3:**
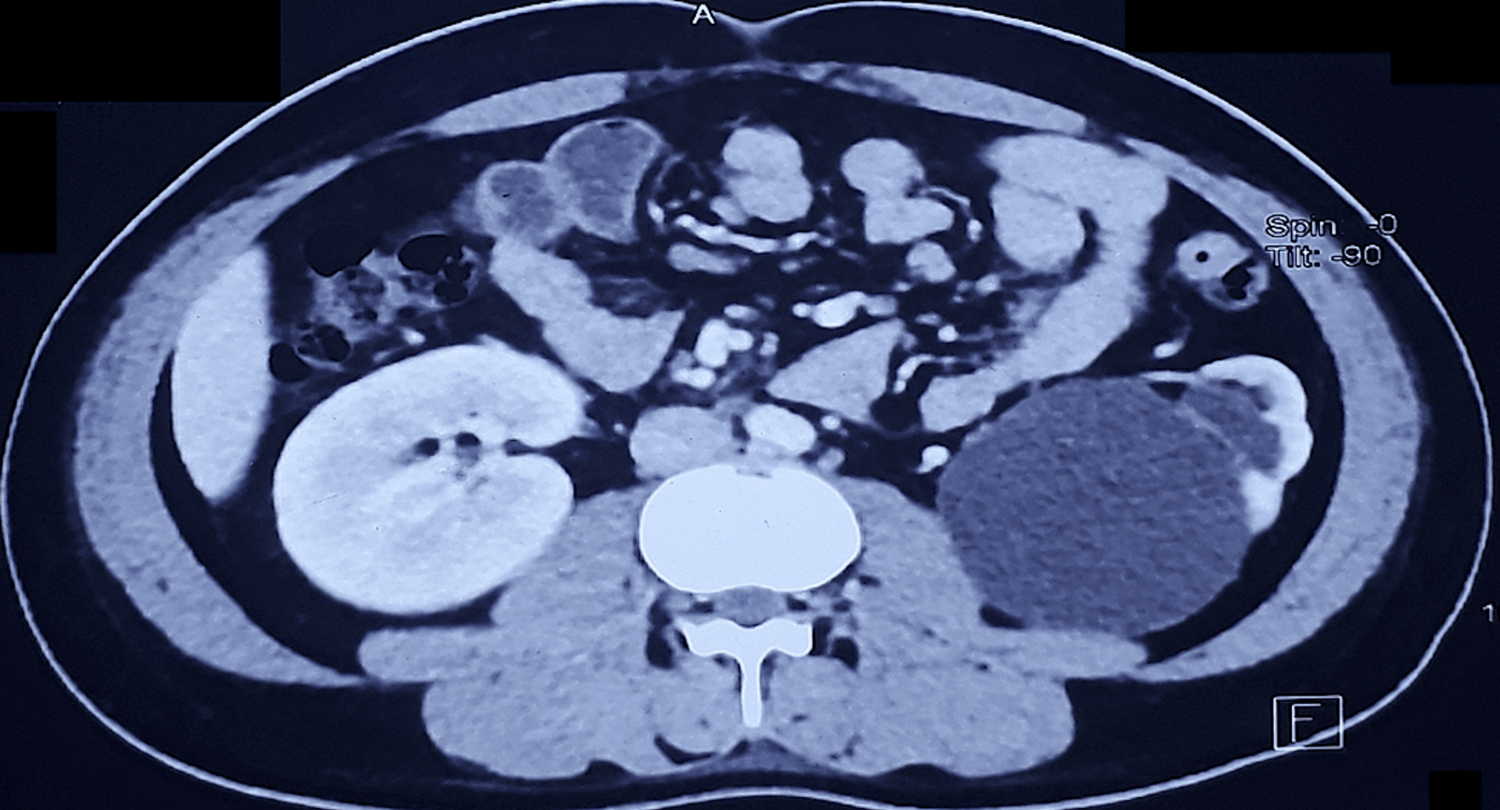
Multidetector CT scan showing a left hydronephrosis with a thinned put parenchyma

**Figure 4:**
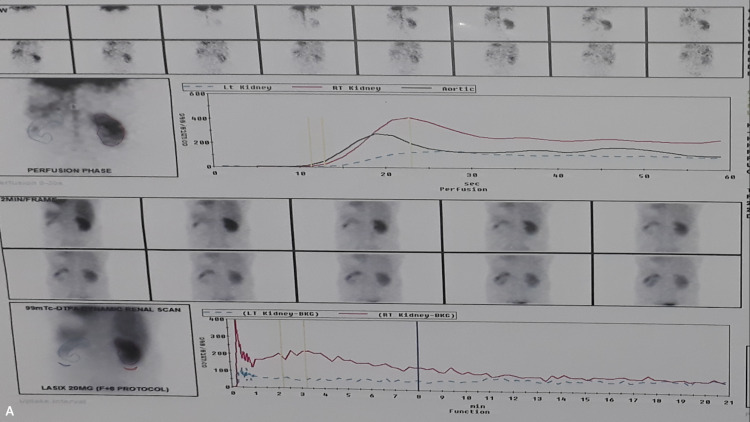
Tc-99 DTPA renogram with diuretic showing decreased functionality and sluggish drainage of the left hydronephrotic kidney Tc-99m DTPA: technetium-99m diethylenetriaminepentaacetate

A 27-year-old male presented with complaints of intermittent left flank pain for six months. An ultrasound revealed gross hydronephrosis on the left side with thinning renal parenchyma. His serum creatinine was within normal limits. A CT urography was done, which revealed a dilated PCS with abrupt narrowing of the UPJ suggestive of a UPJO. There was significant parenchymal thinning and delayed contrast excretion. A diuretic scintigraphy scan done showed a differential function of 20% on the left side (left kidney glomerular filtration rate (GFR) of 12.2 ml/minute) with a dilated PCS and sluggish drainage of contrast (time to peak 20.6 minutes). He underwent a robot-assisted left Anderson-Hynes dismembered pyeloplasty. Intraoperatively, the left testicular vein was seen crossing the UPJ, causing the obstruction. The procedure was uneventful, with a total console time of 65 minutes. The patient was discharged on day three, and the stent was removed after four weeks. A six-week follow-up DTPA scan showed improvement in drainage function with a time to peak T1/2 of 13.5 minutes (Figures 3, 4).

Case 3

A 38-year-old female presented with complaints of right flank pain radiating to the back for six months. Serum creatinine was within normal limits, and ultrasound revealed a staghorn stone on the right side. She underwent a contrast-enhanced CT scan, which revealed a 3.3 x 2.2 cm staghorn calculus on the right side with a mildly dilated right upper ureter due to extrinsic compression caused by the right ovarian vein in its mid part. After ensuring a sterile urine culture, a percutaneous nephrolithotomy (PCNL) was done with complete stone clearance. The procedure and perioperative period were uneventful, and the stent was removed after three weeks. She was advised to get a DTPA scan to characterize the drainage pattern; however, she was lost to follow-up.

Case 4

**Figure 5:**
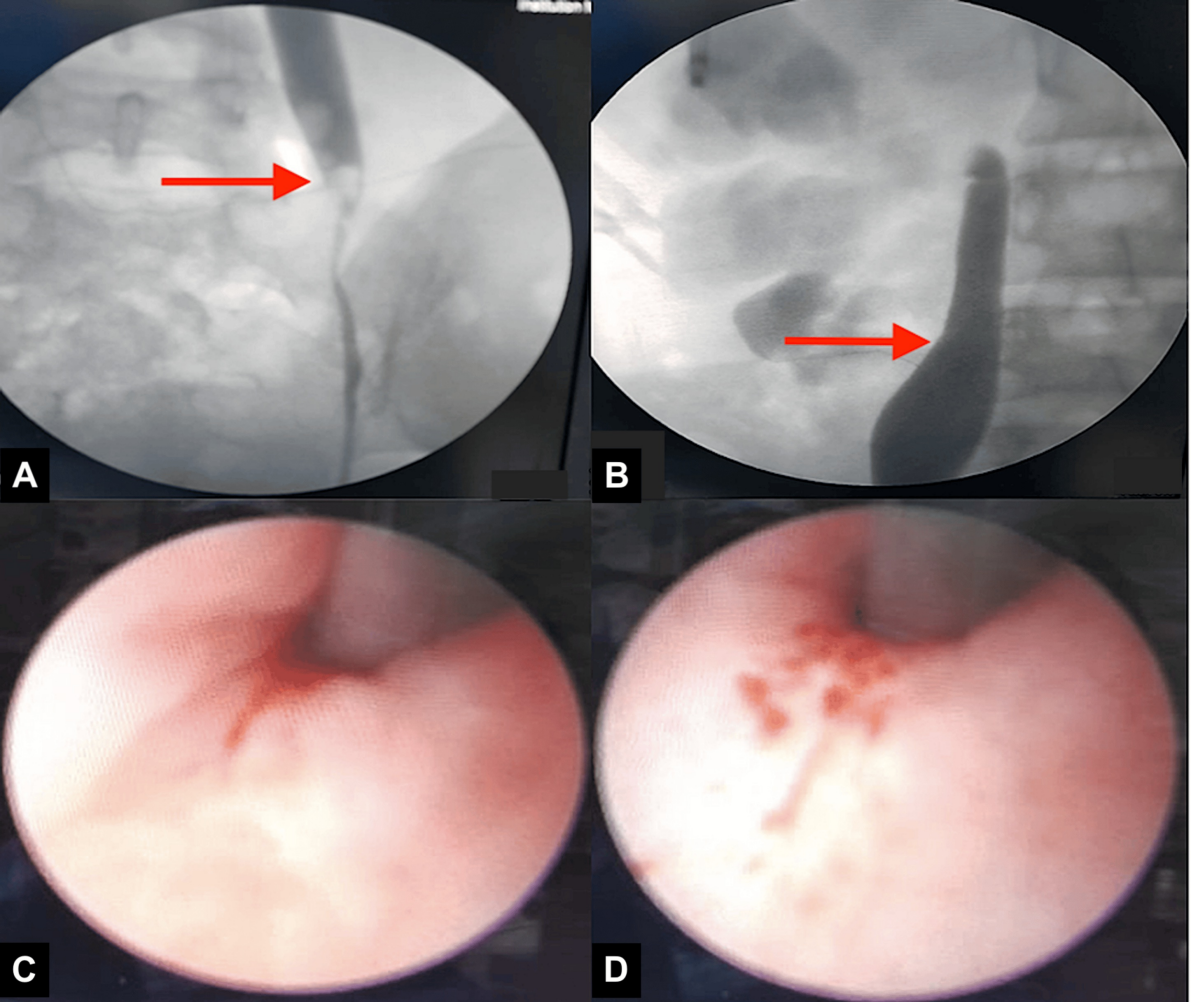
Bilateral retrograde pyelography (RGP) (A) Left ureter narrowing at L5/S1 with upstream dilated ureter and pelvicalyceal system; (B) dilated upper right ureter. Ureteroscopic view of the narrowing at the level of the iliac crossing on (C) the left side and (D) the right side.

A 19-year-old young female with a history of bilateral PCNL done elsewhere one year prior presented to us with complaints of intermittent bilateral flank pain for the past three months. The physical examination was unremarkable. Blood investigations revealed a normal serum creatinine. Urine culture was sterile. In view of gross bilateral hydroureteronephrosis on ultrasound, a CT urography was done, which revealed abrupt narrowing of the ureteral lumen at the level of the common iliac artery crossing (L5/S1 vertebral level) bilaterally with gross upstream hydroureteronephrosis and thinning of parenchyma. A DTPA scan done revealed bilateral functioning kidneys (split function: right 42% and left 58%) and sluggish drainage. An MRI of the abdomen with urography was done to better characterize the lesion. Abrupt narrowing of the lumen suggestive of a stricture was noted at L5/S1 levels bilaterally due to external compression by bilateral common iliac arteries. The patient underwent bilateral ureteroscopy and bilateral retrograde pyelogram to better characterize the lesion. Ureteroscopy revealed narrowing of the lumen at the level of crossing of the iliac vessels bilaterally, with normal mucosa. In view of symptoms and upstream dilatation, 6 Fr stents were placed bilaterally. On the six-week telephonic follow-up, the patient is doing fine and is symptom-free. Due to the ensuing lockdown, the patient is unable to follow up and is planned for a diuretic scan following stent removal to better understand the drainage pattern and possible definitive management at a later stage (Figure 5).

Case 5

**Figure 6:**
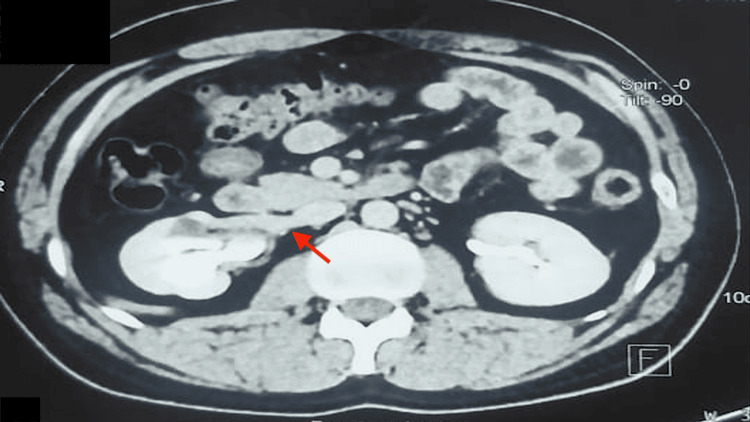
Multidetector CT scan showing compression of the right ureter by the crossing tributary of IMV, causing upstream hydroureteronephrosis IMV: inferior mesenteric vein

**Figure 7:**
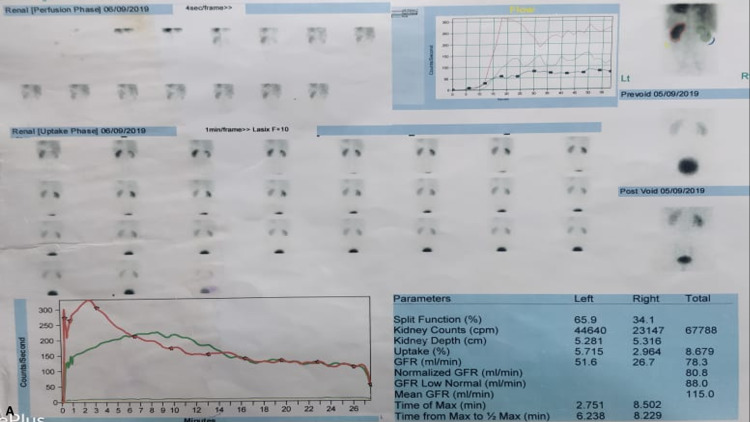
Tc-99 DTPA renogram with diuretic showing reduced parenchymal function with unobstructed drainage of the left hydronephrotic kidney Tc-99 DTPA: technetium-99m diethylenetriaminepentaacetate

A 26-year-old man presented with complaints of right flank pain for six months. He had a history of right ureteroscopic lithotripsy (URSL), conducted three years ago. The physical examination was unremarkable. A CT scan revealed narrowing in the right ureter at the L3 level, accompanied by upstream hydroureteronephrosis. At the point of narrowing, an unnamed tributary arising from the IVC was visualized crossing the ureter. A DTPA scan showed decreased uptake in the right kidney, with a split function of 34%, and unobstructed drainage across the narrowing, with a T1/2 of 8.2 minutes. The patient underwent ureteral stenting with relief of symptoms. Further management will be done after reassessment of the drainage pattern and level of hydroureteronephrosis after stent removal (Figures 6, 7).

## Discussion

Normal anatomical course of the ureter

The ureter is 25-30 cm long, running in the retroperitoneum from the renal pelvis to the bladder. Along its way, it is crossed by several vascular structures. At the level of the inferior pole of the kidney, the abdominal part of the ureter is crossed by the gonadal vessels (artery and vein) from the lateral to the medial side (the famous "bridge over water"). Next, the inferior mesenteric artery and vein, during their lateral course, come really close to the ureter on its medial side. At the level of the pelvic brim, the ureter crosses over the iliac vessels from lateral to medial (over the common iliac artery or the origin of the external iliac artery) [2].

**Figure 8:**
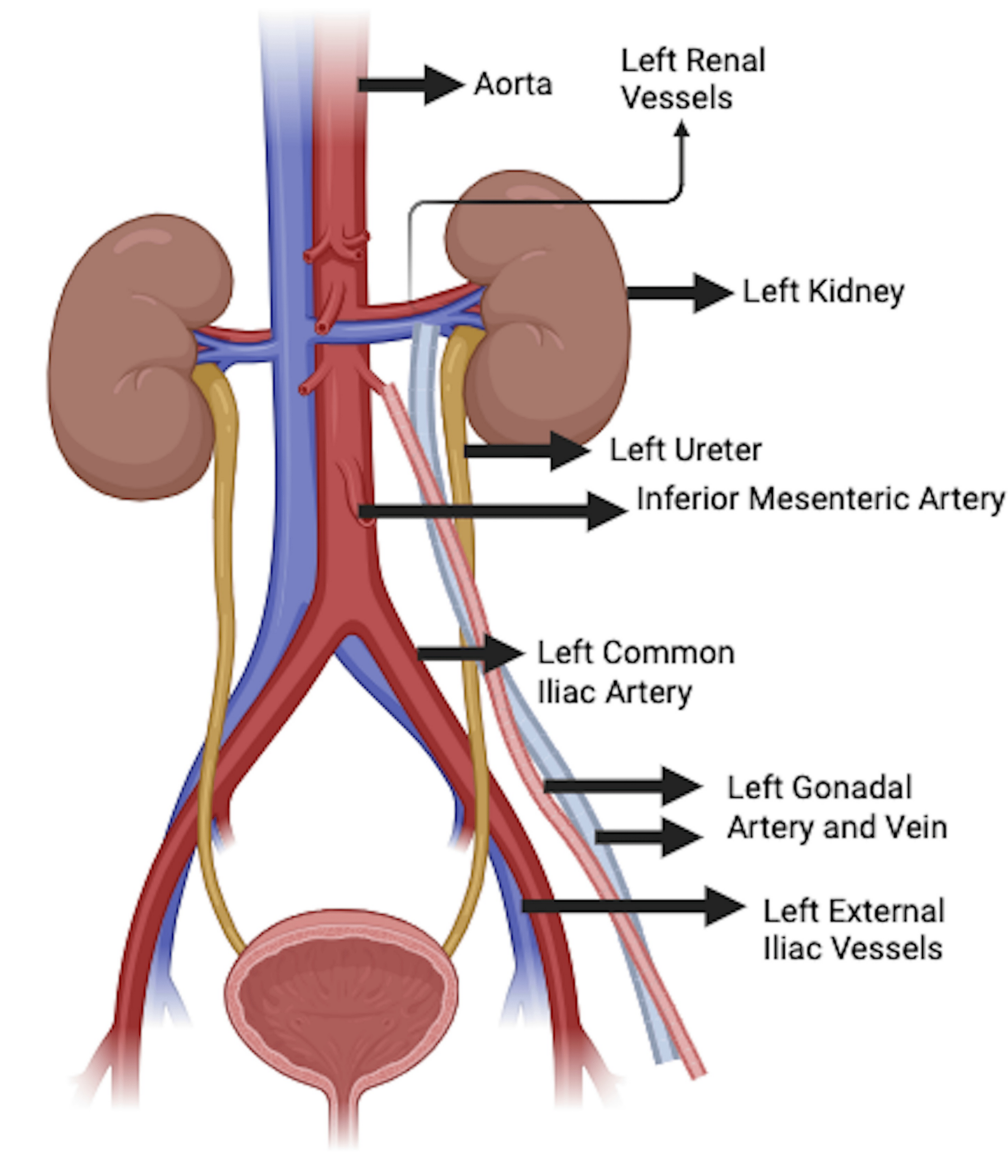
Schematic illustration describing the normal course of the left ureter and the crossing of vascular structures Illustration by Dr. Harkirat Singh Talwar

Other vascular relations of the ureter include the sigmoid vessels anterior to it on the left side, internal iliac vessels posterior to the pelvic part of the ureter, ovarian vessels in the infundibulo-pelvic ligament anterolateral to it at the level of the pelvic inlet, and uterine vessels being crossed by the ureter from below before it enters the bladder (the other famous "bridge over water"). Being a hollow tube, the ureter can be compressed by the various vascular structures it is crossed by, either normal or abnormal variations (Figure 8) [3].

Gonadal vessels and the ureter

Gonadal vessels have a very intimate relation to the ureter. Compression of the ureter by the testicular vein has been previously reported in the literature, known as the "testicular vein syndrome." It is a rare entity, with only eight cases described thus far [4], and ours is the ninth case (Case 2). Hamidi [4] reviewed the testicular vein syndrome and found four such cases on the right side and four cases on the left side. The first case was reported by Mellin and Madsen in 1975 [5]. Most of these cases had intermittent flank pain as the mode of presentation and had a history of renal stone disease. Intravenous urography (IVU) and CT urography were crucial tools in diagnosing the condition. The crossover of the testicular vein is very close to the pelviureteric junction, which may lead to misdiagnosis as a case of UPJO, particularly in younger patients, as exemplified by our Case 2, where the condition was diagnosed intraoperatively.

The treatment of this condition depends on the symptoms and the degree of dilatation of the upper tracts. Symptomatic patients and/or those with marked or severe hydronephrosis require surgical intervention. The previously reported cases have described transection of the vein, ureterolysis, resection of the vein, and excision of the atretic segment, with ureteroureterostomy as one of the various modalities depending on the involved pathologic process [6]. In the index case of our study, this entity was diagnosed intraoperatively with the left testicular vein as the crossing vessel very close to the UPJ. Hence, an Anderson-Hynes type of pyeloplasty was performed with preservation of the testicular vein. This was achieved through a minimally invasive approach using the da Vinci platform.

The counterpart, known as "ovarian vein syndrome" (OVS), is a well-described entity in the literature in which a pathologically altered ovarian vein (thrombosis, phlebitis, thrombophlebitis, or ectasia) may indent onto the ureter, causing upstream pressure changes. This is in contrast to the testicular vein syndrome, in which the normal testicular vein is the culprit. The first case was described in 1964 by Clark [7]. The various etiologies behind the dilatation of the ovarian vein include pregnancy, pelvic congestion, and even hormonal imbalance [8,9]. Most commonly, it is encountered on the right side and causes a clinically insignificant proximal hydroureter in the majority of cases. Due to the stasis of urine and changes in backpressure, hydronephrosis, renal stone disease, and pyelonephritis are well-documented associated features of OVS. A multidetector CT scan remains the primary diagnostic tool. Our index patient (Case 3) presented with a large staghorn calculus on the right side, with an ovarian vein compressing the ureter at the L5 level with mild upstream dilatation. Stone clearance was achieved by PCNL. The patient was asymptomatic post stone clearance, and a follow-up ultrasound at three months showed mild dilatation of the PCS. Hence, observation for the OVS is the management of choice in such asymptomatic cases. The definitive treatment of this entity in symptomatic patients includes either ovarian vein embolization [10] or ovarian vein ligation with or without ureteroureterostomy [11].

Inferior mesenteric vessels and the ureter

The IMV lies to the left of the artery and is in medial relation to the ureter during its curved course. The vein ascends along the psoas muscle to join the splenic vein. No case has been reported in the literature thus far of a ureteral compression syndrome being caused by the IMV. To the best of our knowledge, our index case (Case 1) is the first ever case to be reported. This is highly unusual, as both structures are quite parallel to each other. Nevertheless, in our case, the ureter was compressed between the IMV and the psoas muscle. A very characteristic finding was the association of flank pain with eating. The pain increased in intensity postprandially. This can be explained by an increase in the postprandial venous outflow, causing further impingement of the vein onto the ureter; besides, it reconfirms the diagnosis. Although endoscopic stenting was used as a temporary measure for pain relief and also with an aim to straighten out any kink in the ureter if present, the proximal hydroureter and hydronephrosis were persistent even after stent removal. Also, the patient experiences intermittent episodes of postprandial right flank pain. He is planned for a ureteroureterostomy preserving the IMV. Similarly, Case 5 revealed the presence of an unnamed tributary of the IVC at its narrowing point, likely due to compression. Thus, once again, a multidetector CT scan clinched the diagnosis, and the treatment of this rare entity depends on the symptomatology, with ureteroureterostomy being reserved for symptomatic patients and/or those with significant upstream hydroureteronephrosis. Thus, although veins are low-pressure systems, compression of other hollow viscera, like the ureter, is plausible. The physiology may be akin to crossing veins, resulting in UPJO, either due to direct compression or by kinking and fibrosis.

Iliac vessels and the ureter crossing

Apart from the UPJ and the VUJ (vesico-ureteric junction), the third physiological narrowing in the ureter is at the level of the iliac vessel crossing (L5/S1). A thorough search of the literature revealed only one report citing this unusual clinical scenario [12]. Overpressure by a normal iliac artery (common/iliac) over a fixed ureter can cause ureteral obstruction and changes in back pressure. This might or might not result in symptoms. In a study by Rathi et al. [13] to ascertain the cause of ureteral dilatation on IVU, it was found that three out of the 18 cases had an extrinsic compression of the ureter by the common iliac artery. Although most of the cases are clinically insignificant, this condition may present with renal stones or pyelonephritis due to stasis of urine and back pressure. Our index case (Case 4) also had a history of renal stone disease bilaterally, for which she underwent PCNL elsewhere. The condition was diagnosed on CT urography and confirmed by an MR urography. The strictures were attributed to compression by the crossing external iliac artery. Since they were very short segment strictures (1 cm), a decision was made to manage them endoscopically using holmium laser technology. Aneurysms of the iliac arteries can impinge on the ureter and cause obstruction, but it is rare for a normal caliber artery to do so. Although branches of the internal iliac artery, such as the obliterated umbilical artery, have been shown to be causes of lower ureter obstruction by vascular structures, as shown in the series by Read and Devine [14] and Grifoni et al. [15], this is only the second case to be reported of ureteric stricture caused by a common iliac artery. Additionally, this is the first case to present bilaterally. Management of these cases poses a challenge. Short segment strictures (<2 cm) can be tackled by ureteroscopic endoureterotomy, albeit there are always chances of recurrence. Additionally, the adjacent pulsating artery requires considerable expertise in performing the procedure. Strictures >2 cm require exploration, transection of the stricturous segment, and reconstruction with preservation of the offending vessel.

The limitations of the present study include that it is a retrospective study. Additionally, one case was lost to follow-up, and in the other cases, the definitive management of ureteric reconstruction is yet to be undertaken. The authors wish to follow up as and when the management is complete and the data are available. As a single-center, retrospective case series, our inclusion relied on available records of hydroureteronephrosis cases with documented vascular compression. We recognize that not all eligible cases may have been captured, and this may introduce selection bias. To mitigate this, we applied strict inclusion and exclusion criteria and conducted two independent record reviews to ensure consistency and accuracy. Data were abstracted from imaging and clinical reports initially generated for routine care, rather than research, which raises the possibility of incomplete or inconsistent documentation. We used a standardized data abstraction form and predefined variable coding to reduce misclassification.

## Conclusions

Extrinsic vascular compression of the ureter, although rare, represents a clinically significant, often under-recognized cause of proximal hydronephrosis and hydroureteronephrosis. Unlike classical UPJO or retrocaval ureter, which are well-known urologic entities, this series highlights unusual compressors such as engorged testicular and ovarian veins, the IMV, and even the external iliac artery. These vascular compression syndromes may be symptomatic, manifesting as flank pain, hematuria, urolithiasis, or pyelonephritis, or clinically silent, discovered incidentally on imaging. Therefore, in cases of unexplained hydronephrosis, especially when imaging excludes intrinsic lesions, venous-phase, contrast-enhanced CT urography is essential to identify possible vascular impingement.

Management should be tailored to the severity of symptoms and the degree of obstruction. Temporary stenting or nephrostomy may be necessary for acute relief in complicated cases (e.g., pyelonephritis, stones). Definitive intervention may involve minimally invasive (endoscopic and laparoscopic ureterolysis +/- reconstruction). While a retrospective series is limited in statistical power, our findings underscore that vascular compression should be included in the differential diagnosis for proximal urinary obstruction, even in asymptomatic cases. Early recognition enables targeted, minimally invasive treatment strategies, thereby improving outcomes and preserving renal function.
